# Read, spot and translate

**DOI:** 10.1007/s10590-021-09259-z

**Published:** 2021-04-04

**Authors:** Lucia Specia, Josiah Wang, Sun Jae Lee, Alissa Ostapenko, Pranava Madhyastha

**Affiliations:** 1 Imperial College London, London, UK; 2 University of Pennsylvania, Philadelphia, USA; 3 Worcester Polytechnic Institute, Worcester, USA

**Keywords:** Multimodal machine learning, Multimodal machine translation

## Abstract

We propose multimodal machine translation (MMT) approaches that exploit the correspondences between words and image regions. In contrast to existing work, our referential grounding method considers *objects* as the visual unit for grounding, rather than whole images or abstract image regions, and performs visual grounding in the *source* language, rather than at the decoding stage via attention. We explore two referential grounding approaches: (i) implicit grounding, where the model jointly learns how to ground the source language in the visual representation and to translate; and (ii) explicit grounding, where grounding is performed independent of the translation model, and is subsequently used to guide machine translation. We performed experiments on the Multi30K dataset for three language pairs: English–German, English–French and English–Czech. Our referential grounding models outperform existing MMT models according to automatic and human evaluation metrics.

## Introduction

**Fig. 1:**
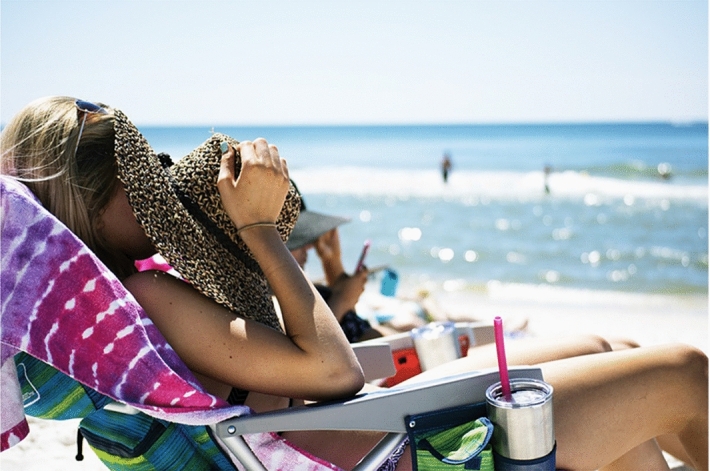
The word * hat* in the description “Woman covering her face with her hat.” is ambiguous when translating into German. The image is needed to enable the selection of the correct word, * Hut* (summer hat), rather than *Mütze* (winter hat)

Multimodal machine translation (MMT) is a research field that aims to enrich textual context with additional modalities (images, videos, audio) for machine translation (MT). The assumption is that context provided by these modalities can help ground the meaning of the text and, as a consequence, generate more adequate translations. This grounding is particularly needed when translating content that is naturally multimodal, such as picture posts on social media, audio descriptions or subtitles. MMT is especially useful when dealing with ambiguous or out-of-vocabulary words. One example is given in Fig. 1, where even a human translator would need to see the image to decide which word to use when translating the ambiguous word *hat* into German (distinction between summer hat *Hut* and winter hat *Mütze*).

**Fig. 2:**
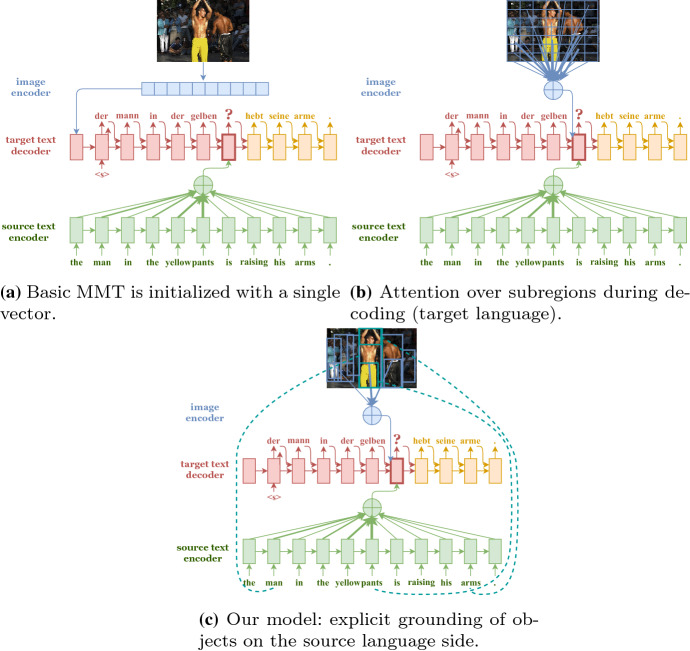
Methods of incorporating images as context in neural MMT architectures. In **a**, the decoder (sometimes encoder) is initialised with a single global vector representation of the image. In **b**, an attention-based model learns to attend to the CNN convolutional layer at each time state of the decoder. Our referential grounding approach in **c** uses object bounding boxes as visual units, grounds object bounding boxes to source words in the encoder (dotted lines), and uses the grounding to guide MT

Existing work on image-based MMT (Specia et al. 2016; Elliott et al. 2017; Barrault et al. 2018) (see Sect. 2), especially neural network approaches, often incorporate images as context either as a single, global vector representation of the whole image (Fig. 2a), or by attending to grid-based representations of different local subregions of the image (Fig. 2b). We argue that such models do not exploit images effectively for MT. A global image representation provides only a summary of the image and is expected to apply equally to the whole text, while MT operates at the word level. For attention-based models, there is a mismatch between the visual unit (equally divided image grid-like subregions) and the textual unit (a word), as the subregions may not correspond to a word or cover multiple words. This makes it hard to learn the correspondence between the textual and visual units during decoding due to a lack of visual consistency, especially when trained on small datasets; any assumed learned correspondences are also hard to interpret since the subregions are not well defined.

**Fig. 3:**
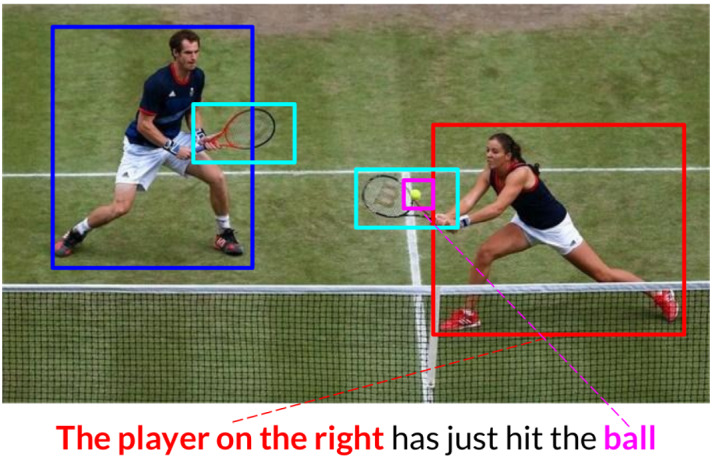
Multimodal correspondences can be used to help guide translation, for example potentially resolving the gender ambiguity of the word * player* such that it can be correctly translated to its feminine form into a gender-marked language

In this paper, we propose new referential grounding approaches to MT where the correspondences between the visual units (object regions) and textual units (source words) are better defined, and can then be used more effectively for translation (Fig. 2c). By *object region*, we mean the depiction of the entity instance from the image as a single, coherent unit. The object instance can be a concrete entity, amorphous ‘stuff’ (*sky*, *cloud*), or a scene (*beach*, *forest*). The main motivation for using objects as a visual unit is that it can potentially result in better and more interpretable grounding. As a motivational example, Fig. 3 shows a case where the ambiguous word *player* can be translated correctly into a gender-marked language (female player) if its correspondence to the correct region in the image is identified.

Our main contributions are:

1. An *implicit referential grounding* MT approach where the model jointly learns how to ground the source language in the object-level image representations and to translate (Sect. 4), and we explore training regimes with and without providing the correspondence as supervision;
2. An *explicit referential grounding* MT approach where object-level grounding is performed on the source side, independent of the translation model, and is subsequently used to guide MT (Sect. 5), and we vary the ways in which the visual information is fused to the textual information; and
3. A strategy that automatically proposes and *aligns objects to words*, which can be used as input for the explicit grounding or potentially as supervision for learning implicit grounding, replacing gold-standard annotations (Sect. 3).

The results of our experiments (Sect. 6) show that the proposed referential grounding models outperform existing MMT models according to automatic evaluation metrics that assess the general quality and lexical ambiguity, and according to manual evaluation where humans assess the adequacy of the translations.

## Related work

*MMT with a single vector* The first approaches for MMT represent images as a single, global vector. These vectors are usually the output of a Convolutional Neural Network (CNN) layer. The layers that have been used are the penultimate layer (Elliott et al. 2015; Caglayan et al. 2017) and the final softmax layer (Madhyastha et al. 2017). The image representation is integrated into the MT models in different ways: (i) by initialising the hidden state of the encoder or decoder (Elliott et al. 2015; Caglayan et al. 2017; Madhyastha et al. 2017); (ii) by element-wise multiplication with the source word annotations (Caglayan et al. 2017); or (iii) by projecting both the image representation and encoder context onto a common space to initialise the decoder (Calixto and Liu 2017). Other methods include re-ranking the output of candidate translations based on the global image representation (Hitschler et al. 2016; Shah et al. 2016; Lala and Specia 2018), and modelling the source sentence and reconstructing the image representation jointly in a multi-task learning setting (Elliott and Kádár 2017; Helcl et al. 2018). A global image vector is, however, limited in that it only captures the gist of the image.

*MMT with attention* most current work on neural MMT utilises an attention mechanism (Bahdanau et al. 2015) on the output of the last convolutional layer of a CNN (Xu et al. 2015). The layer signifies the activation of *K* different convolutional filters on evenly quantised $N \times N$ spatial regions of the image. Methods have been proposed to learn the attention weights for both source text and visual encoders, e.g. via concatenation (Caglayan et al. 2017), combining both attentions independently via a gating scalar (Calixto et al. 2017; Delbrouck and Dupont 2017), applying a hierarchical attention distribution over two projected vectors where the attention for each is learned independently (Libovický and Helcl 2017), and via a doubly-attentive transformer architecture (Helcl et al. 2018). Such attention-based models are closer to our work, although learning attention weights across subregions effectively from limited training data is difficult (Delbrouck and Dupont 2017). Our proposed use of coherent object-level visual units are aimed at alleviating this problem by forming a stronger association between the textual unit and the visual unit.

*MMT with whole objects* Previous work has also explored using object-level regions rather than quantised regions. Huang et al. (2016) detect object category instances in an image, and use the representations for these instances (along with the whole image) to initialise the encoder. Grönroos et al. (2018) extract region segmentations for 80 object categories, and encode the whole image as an 80*D* vector containing the surface area of each category. In both cases, there is no strong association between the words and the regions, and thus object information is not well exploited. In contrast, our referential grounding models make better use of object and text associations.

## Objects as visual units

In what follows we describe how object-level regions are extracted from images and aligned to their corresponding word in the textual description. Before that, we introduce the datasets used in the experiments.

### Data

We build and evaluate our referential grounding MMT models on the **Multi30K** (Elliott et al. 2016) dataset. Each image in Multi30K contains one English (EN) description taken from Flickr30K (Young et al. 2014) and human translations into German (DE), French (FR), and Czech (CS) (Specia et al. 2016; Elliott et al. 2017; Barrault et al. 2018). The dataset contains 29,000 instances in the training set and 1014 in the development set. Each instance comprises an image and its description in four languages (EN, DE, FR and CS). Multi30K is the official dataset in the WMT shared tasks on MMT, with DE introduced in 2016 (Specia et al. 2016), FR in 2017 (Elliott et al. 2017) and CS in 2018 (Barrault et al. 2018). For this paper, we fix EN as the source language and evaluate translations into each of the three target languages (DE, FR and CS).

**Fig. 4:**
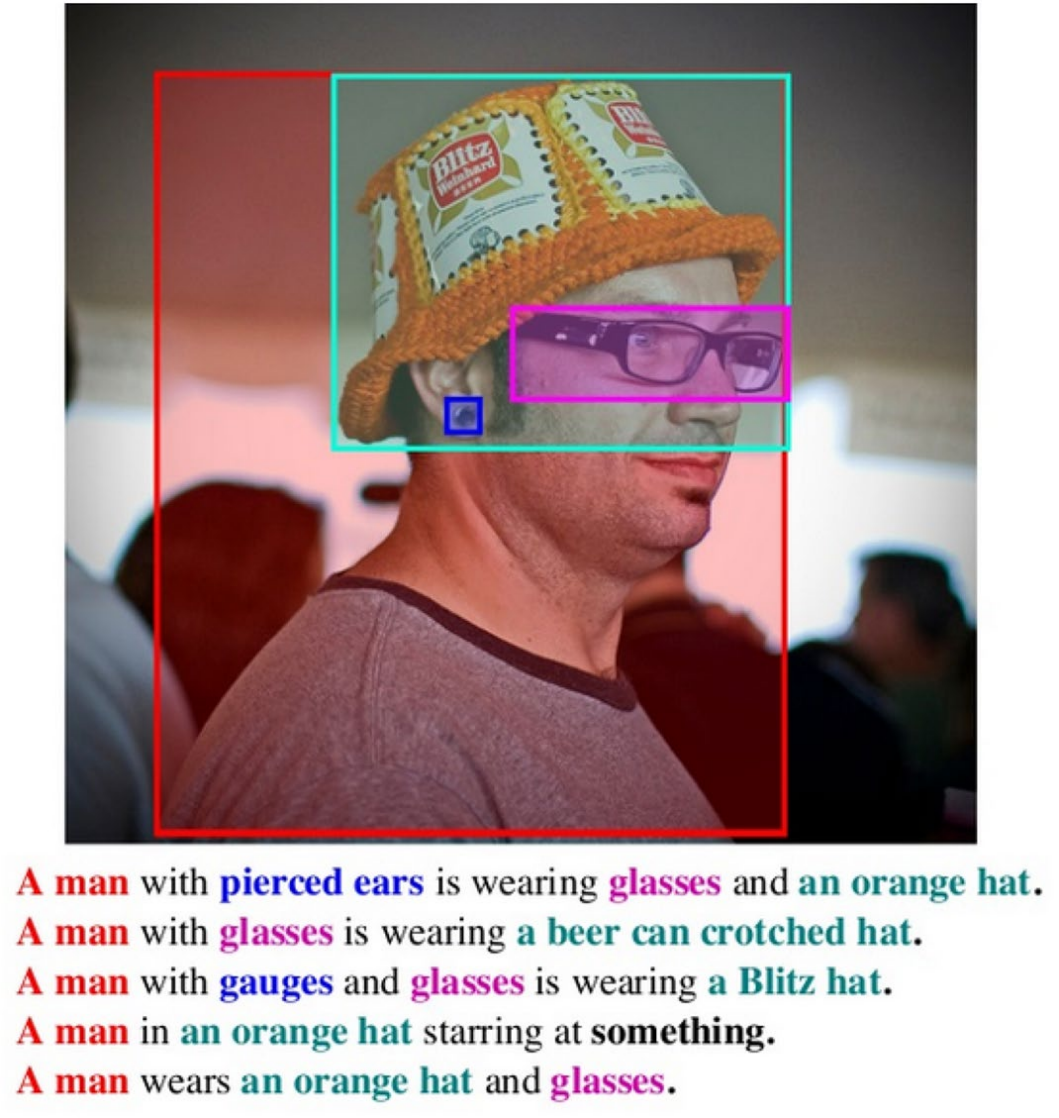
Example of oracle object bounding boxes and object-to-word alignment from Flickr30K Entities

In our experiments, we use automatic and oracle image region annotations (bounding box annotations) and their mapping to words in the text. For the latter, we take the annotations from the **Flickr30K Entities** (Plummer et al. 2015) dataset. In the dataset, each entity mention (noun phrase) in Flickr30K descriptions was manually annotated with a bounding box localisation of the instance(s) depicted in the image. An example is given in Fig. 4, which contains the five original descriptions (the one randomly selected for Multi30K in this case is the fourth). Any entity mention not depicted (without a bounding box) is labelled as non-visual. In Flickr30K entities, each entity mention is also assigned at least one out of eight high-level categories (*person*, *clothing*, *bodyparts*, *animals*, *vehicles*, *instruments*, *scene*, and *others*). These category labels are used in our models in Sect. 5.

### Object bounding boxes

Our referential grounding models rely on object-level information. Therefore, we consider bounding box localisations of an object as “region”; there is scope to use object segmentations instead of bounding boxes in future work.

For our experiments, we are interested in testing the hypothesis that region-specific grounding is beneficial for translation. Therefore, we focus on an **oracle** scenario where object bounding boxes are given by humans for entity mentions in the source description. However, to test the feasibility of our models when this information is not available, for a subset of the proposed models we also experiment with a **predicted** scenario where object bounding boxes for entity mentions are automatically generated by an off-the-shelf object detection tool.

*Oracle object bounding boxes* The oracle scenario aims to evaluate the referential grounding capabilities of the proposed approaches with gold standard bounding boxes for objects, isolating the challenge from having to automatically propose these bounding boxes. For this, we use the bounding box annotations provided by the Flickr30K Entities dataset.

**Fig. 5:**
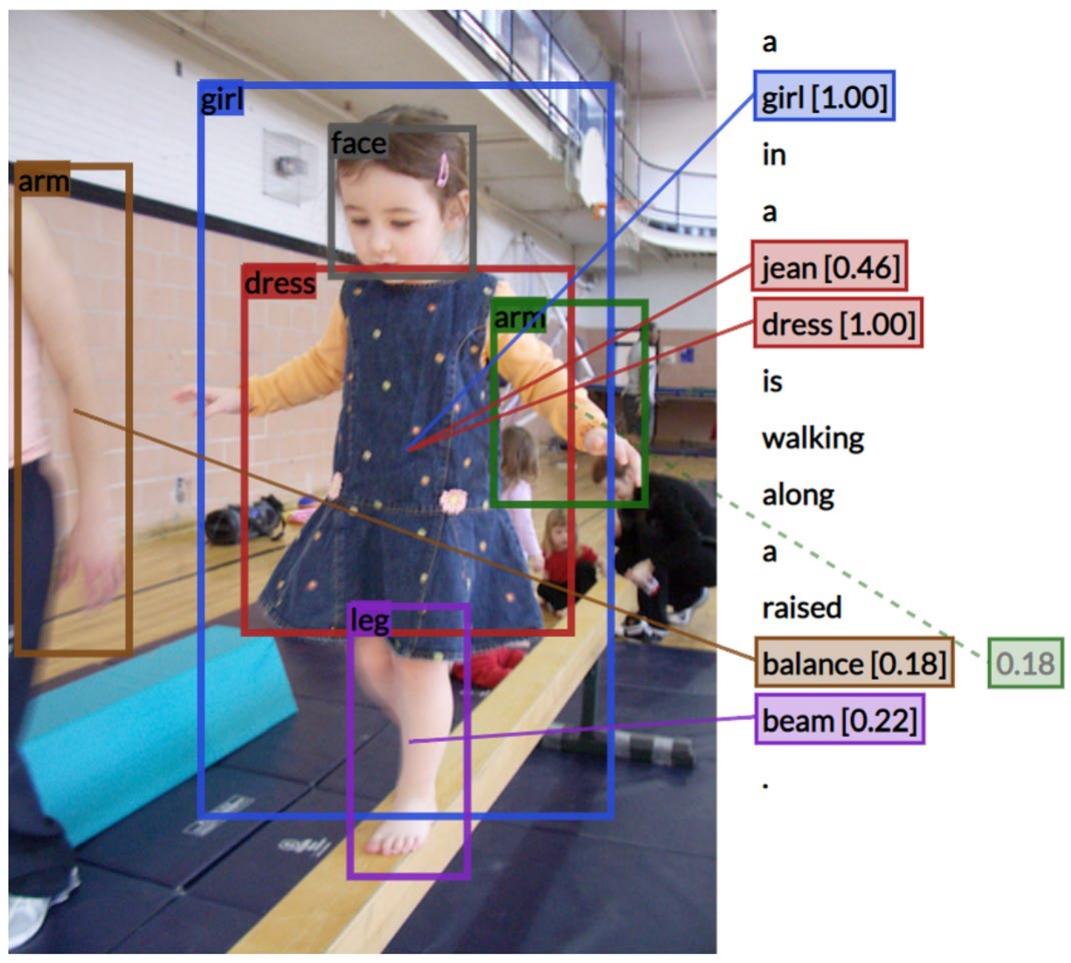
Example output from our object-to-word alignment model using the automatically detected objects

*Predicted object bounding boxes* We use an object detector to produce candidate bounding boxes and object categories. More specifically, we use the Faster R-CNN (Ren et al. 2015) detector pre-trained on the 545 object categories of the Open Images Dataset (V2) (Krasin et al. 2017), with the Tensorflow Object Detection API (Huang et al. 2017).^1^ from https://github.com/tensorflow/models/blob/master/research/object_detection/g3doc/detection_model_zoo.md. We note that these 545 categories mostly contain more fine-grained versions of the 8 very general categories in Flickr30K Entities (e.g. *man, woman, girl, boy, dress*), as shown in the example in Fig. 5.

We note that object-level annotations (be it oracle or automatically predicted) are needed for all region-specific multimodal MT approaches proposed in this paper and described in Sects. 4.2, 4.3, and 5. The only strategy that does not require object-level annotation is the baseline multimodal model in Sect. 4.1.

### Object to word alignment

Some of our approaches require annotations on the correspondence between entity mentions in the source text and their localisation in the image. More specifically, this annotation is required for the training of the supervised co-attention approach in Sect. 4.3, and for both training and decoding of the explicit grounding approaches in Sect. 5.

Here we again explore the **oracle** approach where we take alignments previously annotated by humans for the corresponding bounding boxes also annotated by humans. In other words, we start with the oracle object bounding boxes as described in Sect. 3.2 and use the correspondence annotations as given in Flickr30K Entities. An example is shown in Fig. 4, where the colours indicate the alignment. As our decoder operates at word level, we assume that any content word in the phrase for an entity mention can refer to the depicted object instance.

To test the feasibility of our models in the absence of these oracle alignments, in the case of automatically detected bounding boxes, we also propose an **automatic** alignment, where we infer an alignment for each bounding box. Here we start with the predicted object bounding boxes as described in Sect. 3.2 and infer the connection of these to words in the text. This alignment method is fully unsupervised, i.e. it uses *no* training data from Multi30K or Flickr30K.

More specifically, for each image, we compute the semantic similarity between a word and the category label for each detected bounding box instance. The intuition is that the bounding box instance that is very similar or related to a word is most likely to be the target object. For example, the word *dancer* is similar or related to the category *person*. We represent a word *w* and a detector label *d* (object category) as 300-dimensional CBOW word2vec embeddings (Mikolov et al. 2013). Detector labels comprising multiword phrases are represented by the sum of the word vectors of each in-vocabulary word of the phrase, normalised to unit vector.

We align a word *w* to the detected bounding box instance *d* with the highest cosine similarity *S*(*w*, *d*). If there are multiple bounding box instances with the highest score, we align it to the largest bounding box (most likely to be mentioned). We constrain alignment to nouns to reduce misalignments.^2^ Figure 5 shows an example output from our automatic aligner. Note that we can obtain phrase alignments, e.g. *jean dress*.

We note that the combinations of object bounding boxes and object–word alignment strategies result in two settings (i) a fully oracle-based annotation (object bounding boxes and object–word alignments), where we are able to isolate the grounding capabilities of the model from these two intermediate steps, and (ii) a fully automatic annotation (detected object bounding boxes and automatic object–word alignments), which is a more realistic setting. While other combinations could be possible, they are less appealing. On the one hand, if the bounding boxes are predicted automatically, we cannot rely on oracle object–word alignments, as the detected objects can be different from the ones annotated by humans. On the other hand, while we could combine oracle object region annotations with automatic object-to-word alignments, the outcome of models trained in this way would be less insightful.

## Implicit grounding

We propose two new attention mechanisms for MMT where (i) grounding happens on the source language (Sect. 4.2), and (ii) this process is supervised by examples of aligned word–image region annotations (Sect. 4.3). We start by describing our baseline MMT model (Sect. 4.1).

### Baseline attention-based MMT

As a baseline, we experiment with the standard visual attention approach by Caglayan et al. (2017) and its extension to hierarchical fusion by Libovický and Helcl (2017), which proved effective in their work. These approaches do not use object-level representations but convolutional feature maps, which are believed to capture spacial information in the image that could correspond to image regions (although there are no guarantees that this happens in practice).

The image features for an image *I* are extracted from the last convolutional layer of a 152-layer ResNet (He et al. 2016) as a $14{\times }14{\times }1024$ feature map. In the standard approach to visual attention, given the spatial feature map $\phi (v_{j})$, where $j \in \{1, \ldots , 196\}$ are the flattened feature maps, and the decoder hidden state at time step *i*, $\tilde{\mathbf {h_{i}}}$, an unnormalised attention score $\mathbf {g_{j}}$ is computed as in (1):

1 $$\begin{aligned} \mathbf {g_{j}} &= \mathbf {W^{\top }_{vg}}\tanh {(\mathbf {W_{v}}\phi (v_{j}) + \mathbf {W}_{\tilde{\mathbf {h}}}\tilde{\mathbf {h_{i}}})}, \end{aligned}$$

where $\mathbf {W_{vg}}$, $\mathbf {W_{v}}$ and $\mathbf {W}_{\tilde{\mathbf {h}}}$ are learned parameters.

The attention probabilities, $\beta _{j}$, are computed as a normalised sum over the feature maps, as in (2):

2 $$\begin{aligned} \beta _{j}&= {\text {softmax}}(\{\mathbf {g_{1}}, \ldots , \mathbf {g_{196}}\}). \end{aligned}$$

We then obtain the visual context vector—an attention weighted sum over the feature maps—as in (3):

3 $$\begin{aligned} \mathbf {c_{vi}} &= \sum \limits _{j=1}^{196}{\beta _{j}\phi (v_{j})}. \end{aligned}$$

Caglayan et al. (2017) concatenate the visual context vector $\mathbf {c_{vi}}$ with the standard textual context vector $\mathbf {c_{i}}$ while decoding. We instead follow the hierarchical attention approach from Libovický and Helcl (2017) where a second attention mechanism is constructed over the context vectors. This is done over two steps: (i) a context vector per encoder state is computed separately; and (ii) a weighted sum of the distributions over the *n* encoder states is computed. Formally, after the computation of both $\mathbf {c_{i}}$ and $\mathbf {c_{vi}}$, for a source sentence with *n* words the unnormalised attention score $\mathbf {g^{k}_{hier}}$ for encoder state *k* is computed as in (4)–(6):

4 $$\begin{aligned} \mathbf {g^{k}_{hier}}= & {} \mathbf {W^{\top }_{hier}}\tanh {(\mathbf {W}^\mathbf {k}_{\bar{\mathbf {hier}}}\tilde{c}_{i}^{k} + \mathbf {W}_{\tilde{\mathbf {h}}}\tilde{\mathbf {h_{i}}})} \end{aligned}$$

5 $$\begin{aligned} \gamma ^{k}_{i}= & {} {\text {softmax}}(\{\mathbf {g^{1}_{hier}}, \ldots , \mathbf {g^{n}_{hier}}\}), \end{aligned}$$

6 $$\begin{aligned} \mathbf {c_{fin}}= & {} \sum \limits _{k=1}^{n}{\gamma ^{k}_{i}{\mathbf {W}^\mathbf {k}_{\bar{\mathbf {hier}}}}\tilde{c}_{i}^{k}}, \end{aligned}$$

where $\tilde{c}^{k}_{i}$ is the context vector for the *k*th encoder (i.e., $\mathbf {c_{i}}$ or $\mathbf {c_{vi}}$), and $\mathbf {c_{fin}}$ is used as the final context vector for the decoder.

### Source co-attention

Our first object-level grounding model is designed to align source words to object regions using a co-attention mechanism at encoding time. Let ${\mathbf {V}} = v_{1}, \ldots , v_{m}$ be the *m* object-level regions that have been cropped from the image. The visual representation for each object region, $\phi (v_{i})$, is a 2048-dimensional vector generated as a non-linear transform of the penultimate (pool5) layer of a 152-layer ResNet CNN.

Given these representations, we adapt the co-attention mechanism of Lu et al. (2016) to ground the source words, where the model jointly learns to align these words to the image regions, and to translate them. This is done by first obtaining the affinity matrix ${\mathbf {A}}$ as in (7):

7 $$\begin{aligned} {\mathbf {A}} &= \tanh {({\mathbf {H}}^{\top }W_{a}{\mathbf {V}})}, \end{aligned}$$

where ${\mathbf {H}} \in {\mathcal {R}}^{n{\times }d}$ are the encoder hidden states, ${\mathbf {V}} \in {\mathcal {R}}^{m{\times }l}$ are the object-level image representations, and $W_{a}$ is the bilinear parameter matrix. The image and encoder attention maps are obtained as in (8):

8 $$\begin{aligned} \mathbf {C_{s}}&= \tanh {(\mathbf {W_{cs}}{\mathbf {H}} + (\mathbf {W_{cv}}{\mathbf {V}}){\mathbf {A}}^{\top })}\\ \mathbf {a^{s}}&= {\text {softmax}}(w_{cs}^{\top }\mathbf {C_{s}}), \end{aligned}$$

where $\mathbf {a^{s}}$ computes the source affinity. Similarly, visual affinity $\mathbf {a^{v}}$ is computed as in (9):

9 $$\begin{aligned} \mathbf {C_{v}}&= \tanh {(\mathbf {W_{cv}}{\mathbf {V}} + (\mathbf {W_{cs}}{\mathbf {H}}){\mathbf {A}})}\\ \mathbf {a^{v}}&= {\text {softmax}}(w_{cv}^{\top }\mathbf {C_{v}}). \end{aligned}$$

The expectation is that the model learns the alignment between source words and object regions while learning to translate, i.e. the attention weights indicate this alignment.

We also use hierarchical attention (as described in Sect. 4.1) on top of co-attention such that, at decoding time, the model learns to jointly attend to the source context vector computed using the standard attention and the sum of the source affinity attention and the visual affinity attention from Eqs. (8) and (9).

### Supervised source co-attention

Our second object-level grounding model builds on the one described in Sect. 4.2 by modifying the standard co-attention mechanism into a supervised co-attention mechanism. The learning of the alignment between source words to object regions is therefore done with explicit correspondence annotations as supervision. To do so, we expand the co-attention approach by adding an auxiliary loss to the standard cross-entropy loss. The auxiliary loss penalises cases where the co-attention weights are highest for regions other than the correct one. Inspired by phrase localisation work by Rohrbach et al. (2016), given a correct region *j*, we define the grounding loss as in (10):

10 $$\begin{aligned} {\mathcal {L}}_{grounding}& = -\frac{1}{B} \sum \limits _{b=1}^{B}{\log (\Pr (j{\mid }\mathbf {a^{v}}))}, \end{aligned}$$

where *B* is the number of words per batch and $\mathbf {a^{v}}$ is from Eq. (9). Here, we have explicit correspondences between words in the source language and the regions in the image. For each given source word, the loss is only active if the ground truth has an alignment for it, else it is set to zero.

**Fig. 6:**
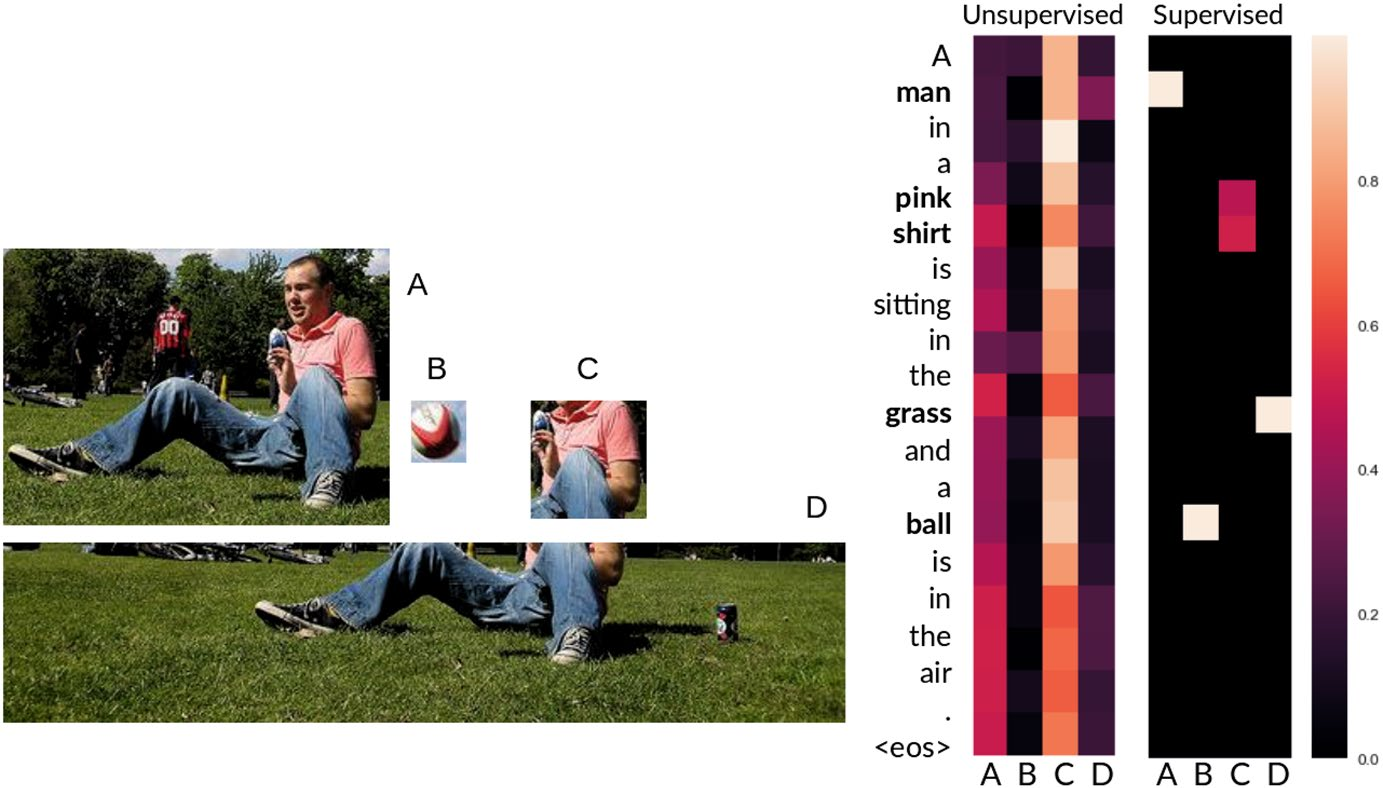
Distribution of attention weights for unsupervised and supervised co-attention mechanism

In Fig. 6 we show an example of attention weights learned for image regions (indicated by letters A–D on the grids) for a source sentence with both the unsupervised and supervised versions of the source co-attention mechanism. The supervised version clearly learns to assign the attention weights to the correct regions for each given content source word.

## Explicit grounding

While attention is a well-established approach, for a dataset as small as ours (30K training instances), we hypothesise that the models may not observe enough instances of similar visual representations with the same textual context for attention to be effective, even in its supervised formulation.

Here we introduce an alternative, two-step approach: first, not only the object regions but also their correspondence (alignments) to words in the source sentence are identified beforehand; second, these correspondences are then fed to the model as additional information further specifying the source words. Previous work has explored specifying word-level information in neural MT for morphological features (Sennrich and Haddow 2016) and for topics (Deena et al. 2017). In both cases, every word was specified with a vector containing the additional information (e.g. POS tags). We follow a similar approach, but our setting is more complex in that we do not have an image region associated to each given word in the sentence (cf. Fig. 5). We focus on specifying nouns only, which are commonly depicted concepts in images. For nouns that do not have a corresponding image region and for all other words in the sentence, such as verbs and function words, we specify them with a vector containing a pre-trained word embedding of the word itself.^3^ For source noun phrases containing more than one word, we specified the head noun only.

As for the content of the additional vector to specify nouns, we experiment with two types of information: (i) specification using object categories; and (ii) specification using CCA projections. In all cases, the two-step process is: we first obtain an alignment of the source words ($\phi (s_{i}) \in {\text {set\, of \, all \, words \, in \,source\, sentence}}\, {\mathcal {S}}$) and the corresponding object category embeddings in the image ($\phi (r_{j}) \in {\text {set \, of \, all \, object \, categories \,for \, a \, given \, image}}\, {\mathcal {R}}$). We then replace the source word embeddings with the corresponding specified embeddings ($\tilde{\phi }(s_{i})$). We describe the process in the following sub-sections.

### Object categories

**Fig. 7:**
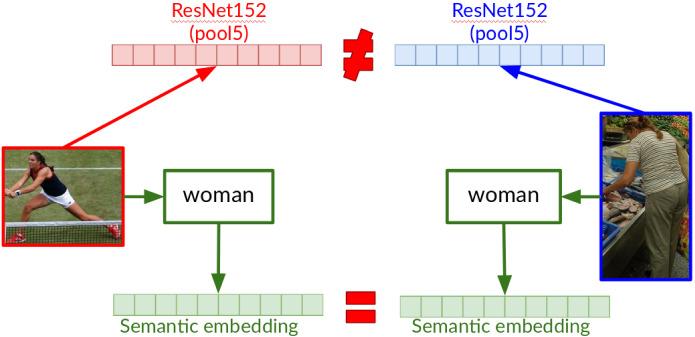
Specification via category embedding versus pool5 features

We specify the words in the source sentence with its aligned object category. As a visual representation for the image region aligned to the word, instead of pool5 features we rely on the word representing the label of the *category* of the object in that region, e.g. *person* or *clothing*. Figure 7 shows a motivational example, where the pool5 visual representation for the two *woman* regions would be very different despite them belonging to the same semantic category. To make the representation more semantically relevant, instead of the word representing the category label itself, we use pre-trained word embeddings for this word. For example, by specification, we expect the visual representations for *woman* and *girl* to be closer than those for *woman* and * dog*.

We further explore two methods to specify visual information in the form of category embedding for words: by *concatenation* and by *projection*.

*Specification by concatenation* Here, the source word embedding is specified with region-grounded information via concatenation:

$$\begin{aligned} \tilde{\phi }(s_{i}) &= [\phi (s_{i});\phi (r_{j})], \end{aligned}$$

where $\phi (s_{i})$ is the source word embedding and $\phi (r_{j})$ is the object-level region information. These are then used to initialise the representations of the words for the encoder bidirectional recurrent units.

*Specification by projection* Alternatively, we learn a linear projection over the region-grounded information:

$$\begin{aligned} \tilde{\phi }(s_{i}) &= \phi (s_{i}) + W\phi (r), \end{aligned}$$

where *W* is a learned affine transformation. Note that, in this setup, the model is learning both $\phi (s_{i})$ and *W*, while the $\phi (r)$ remains fixed. The motivating idea here is that the linear projection is a better combined representation to ground the source embeddings.

### CCA projections

Since the specification involves relating words to image region representations, we evaluate the utility of projecting the image representation such that it is highly correlated with the word representations by using canonical correlation analysis (CCA) (Hotelling 1936). Formally, given paired matrices ${\mathfrak {V}}$ and ${\mathfrak {R}}$, where each row of ${\mathfrak {V}}$ is a visual region and its corresponding word represented by its word embedding ${\mathfrak {R}}$, we generate a linear projection using CCA. We then use these projections to obtain transformed representations of ${\mathfrak {V}}$ as $\mathfrak {V_{cca}}$ and use them as visual features. ${\mathfrak {V}}$ can be either the pre-trained word embedding for the category label of the object (as above) or pool5 features for the object region.

We specify the visual information in the form of CCA projections for words by *concatenation* as:

$$\begin{aligned} \tilde{\phi }(s_{i}) &= [\phi (s_{i}); \phi ({\mathfrak {v}}_{i})_{cca}], \end{aligned}$$

where $\phi (s_{i})$ is the source word embedding and $\phi (\mathfrak {v}_{j})_{cca}$ is the transformed visual representation.

## Experiments and results

We build attention-based sequence to sequence models (Bahdanau et al. 2015) with bidirectional recurrent neural networks with gated recurrent units (Cho et al. 2014) as the encoder and decoder. We use the nmtpytorch tool,^4^ with the following settings: early stop by Meteor (Lavie and Agarwal 2007) (max 100 epochs), selection of best model according to Meteor, beam size = 6, batch size = 64, Adam (Kingma and Ba 2014) as optimiser, word embedding dimensionality = 256, and tokens rather than sub-word units. Experiments with BPE (Sennrich et al. 2016) segmentation on monomodal models did not lead to significant translation quality improvements according to automatic evaluation metrics. In addition, the alignment between subwords and object regions would have been harder than the token-level alignment.

For category embeddings and CCA representations we use fasttext 300-dimensional pre-trained word embeddings (Bojanowski et al. 2017). In the results reported for explicit alignments we specify only head nouns for which an alignment exists to a region in the image, and use the pre-trained embeddings of the words themselves for the remaining words. Table 1 summarises the results for the following models, using BLEU (Papineni et al. 2002) and Meteor, where the latter is the official metric used for this task (following from the MMT shared tasks):

- **Text-only**: Standard NMT baseline without visual information.
- ** SubrAttention**: Standard visual attention over image subregions at decoding time (Sect. 4.1) with hierarchical fusion.
- **CoAttention**: Co-attention over image regions (pool 5 features for objects) and source words (Sect. 4.2).
- **SupCoAttention**: Supervised co-attention over image regions (pool 5 features for objects) and source words (Sect. 4.3).
- **ExplicitProj**: Projection of category embedding information $\mathbf {E_{obj}}$ (Sect. 5.1).
- **ExplicitConc**: Concatenation of category embedding information $\mathbf {E_{obj}}$ and learned word embeddings (Sect. 5.1).
- **ExplicitCCA**: Concatenation of $\mathbf {V_{cca}}$ (pool 5 features for objects) and learned word embeddings (Sect. 5.2).

**Table 1:** Performance of models using oracle object region annotations and alignments, according to Meteor

Systems	EN–CS	EN–DE	EN–FR
Best WMT16	–	53.20	–
Text-only	28.90	**57.35**	74.09
SubrAttention	28.84	55.45	73.31
CoAttention	30.37	57.15	**75.85**
SupCoAttention	30.34	56.48	75.10
ExplicitProj	** 30.63**	57.05	75.02
ExplicitConc	** 30.61**	57.26	75.17
ExplicitCCA	30.52	57.12	75.34
Automatic annotations			
ExplicitProj	30.58	56.96	74.89
ExplicitConc	30.45	57.06	75.05

Results are average of three runs with different seeds. The first row indicates the best system for EN–DE, the only language tested on this test set at WMT16 (Specia et al. 2016). For comparison, the bottom two rows show variants of the two well-performing models where both the object region and alignment annotations are generated automatically

According to Table 1, the proposed multimodal models outperform text-only counterparts for EN–CS and EN–FR, and the standard multimodal approach SubrAttention for all language pairs. We also show that this is the case for variants of two of our best performing models using automatic object detections and object–word alignments in the last two rows of the table. The comparison against automatic object region and alignment annotation is more applicable for ** explicit** grounding models since in implicit grounding alignments are not always needed (they are not needed by CoAttention or SubrAttention). While more experiments on other model variants could be done, the focus of this paper is on showing that object-level information is beneficial for multimodal MT, rather than making a case for the quality of the automatic annotations. However, we posit that the performance of state-of-the-art object detection (e.g., Redmon and Farhadi (2017)) and object–word alignment (e.g., Wang and Specia (2019)) approaches would allow them to be used for this purpose without performance degradation.

While the automatic metric results are generally positive, it has been shown in the WMT shared tasks on MMT (Elliott et al. 2017; Barrault et al. 2018) that automatic metrics can fail to capture nuances in translation quality such as those that we expect the visual modality to help with, which—according to human perception—lead to better translations. This may be particularly the case for EN–DE, where rich morphology and compounding may result in better translations, even though these do not match the reference sentences. Therefore, we also present to two additional evaluations: (i) an automatic evaluation metric on the accuracy of translating ambiguous words only, and (ii) manual evaluation on the adequacy of the translations.

### Lexical ambiguity evaluation

One motivation for incorporating multimodality into MT is that visual features could potentially help disambiguate ambiguous words (Elliott et al. 2015). Thus, the question we ask is whether our MMT models can correctly translate a specific set of ambiguous words in the context of a sentence and image. At the WMT18 shared task on MMT, Barrault et al. (2018) evaluated systems using the Lexical Translation Accuracy (LTA) metric (Lala and Specia 2018), which assesses the disambiguation performance of MMT systems at word level. More specifically, LTA measures how accurately a system translates a subset of ambiguous words found in the Multi30K corpus. A word is said to be ambiguous in the source language if it has multiple translations (as given in the Multi30K training corpus) with different meanings. The subset of ambiguous words in context was created using a semi-automatic process, starting with word alignment to build dictionaries, followed by manual checking of the dictionaries to keep only those words that are actually ambiguous. At test time, a lexical translation is considered correct if it matches exactly the (lemmatised) word aligned to it in the reference test set. Our test set of 1000 sentences contains 1708 such words for EN–DE, 1298 for EN–FR, and 249 for EN–CS. In this paper we use a variant of the LTA methodology: in addition to rewarding cases where the correct translation is found ($+1$), we penalise cases where an incorrect translation is found ($-1$), i.e. a possible translation with a different meaning is generated. If no correct or incorrect translation is found, no reward or penalty is applied. Table 2 shows that all multimodal models are better than their text-only counterpart at translating ambiguous words.

**Table 2:** Performance of models using oracle object annotations and alignments according to LTA

Model	EN–CS	EN–DE	EN–FR
Text-only	10.44	37.00	53.62
SubrAttention	10.84	37.82	53.62
CoAttention	12.45	38.06	**55.16**
SupCoAttention	13.25	37.47	**55.16**
ExplicitProj	13.65	**38.41**	54.08
ExplicitConc	12.85	38.06	53.78
ExplicitCCA	**14.06**	38.17	54.08

### Manual evaluation

For manual evaluation, we randomly sample 50 source sentences to form pairs of instances containing the text-only baseline and one of five multimodal models, where translations differ: SubrAttention, CoAttention, SupCoAttention, ExplicitProj (very similar translations to ExplicitConc), and ExplicitCCA. We then ask a human translator for each language to judge each pair and select the translation that is better at conveying the meaning of the source sentence, given the corresponding image, i.e. to judge adequacy, as in the WMT MMT shared task. In Table 3 we show the proportion of times each model is better than text-only variant. Once again, all our multimodal models are better than their text-only counterparts in more than half of the cases, with CoAttention and ExplicitCCA performing the best. The benefit of multimodality in the standard SubrAttention approach is less prominent. Examples of where multimodal models were judged better at preserving the meaning of the source text can be seen in Table 4. Here we take examples from **ExplicitCCA** for all languages.

**Table 3:** Proportion of times each multimodal model is better than its text-only counterpart at preserving the meaning of the source text

Text-only versus	EN–CS	EN–DE	EN–FR
SubrAttention	0.67	0.38	0.55
CoAttention	0.60	**0.89**	0.60
SupCoAttention	0.67	0.63	0.63
ExplicitProj	0.67	0.67	0.63
ExplicitCCA	**0.88**	0.63	** 0.88**

**Table 4:** Qualitative examples comparing text-only NMT and multimodal models. We show the source (SRC), text-only MT (NMT) and the multimodal model **ExplicitCCA** (MMT)

EN–FR	
	
	SRC: A man on a tag line going into the water.
	NMT: Un homme sur une ligne de métro en train de marcher dans l’eau.
	*(A man on the* *metro line walking* *to the water.*)
	MMT: Un homme sur une **ligne de sable allant** dans l’eau.
	*(A man on the* ***sand line going*** *into the water.)*
	
	SRC: A large group of people of various ages and genders sit outside together.
	NMT: Un grand nombre de personnes de différents âges et des accessoires sont assis ensemble.
	*(A large number of people of different ages and* *accessories* *sit together.)*
	MMT: Un grand nombre de personnes de différentes áges et **d’autres** sont assis ensemble .
	*(A large number of people of different ages and* ***others*** *sit together.)*
EN–DE	
	
	SRC: A man in a gray shirt jumps over the top of a sand dune in the desert .
	NMT: Ein mann in einem grauen hemd springt über das dach einer sanddüne .
	*(A man in a grey shirt is jumping over* *the roof* *of a sand dune.)*
	MMT: Ein mann in einem grauen hemd springt über **die spitze** einer sanddüne in der wüste.
	*(A man in a grey shirt is jumping over* ***the peak*** *of a sand dune in the desert.)*
	
	SRC: A fox terrier leaps after a ball.
	NMT: Ein metzger springt nach einem ball.
	*(A* *butcher* *jumps for a ball.)*
	MMT: Ein **terrier** springt nach einem ball.
	*(A* ***terrier*** *jumps for a ball.)*

In both cases we also show the back-translation into English for clarity. Underlined words represent translation errors, while bold face words, the correct (or better) version

### Oracle versus predicted regions

Thus far we have shown results where the oracle bounding boxes and object–word alignments are used. In the implicit grounding models this is not a major issue given that the alignments are only needed at training time. For the explicit grounding models, however, this information is also needed at test-time. Therefore, we also report results using the predicted objects (i.e. object detections) and object–word alignments. The results, shown in the bottom two rows of Table 1, indicate that there are no significant differences in performance.

## Conclusions

We proposed referential grounding approaches for MMT that use clearly defined correspondences between a source word and an object in the image to guide translation. We showed that MMT models using such groundings at object-level can better exploit image information, leading to better performance, especially when translating challenging cases such as ambiguous words. In future work we will investigate ways to further improve our image segmentation and object–word alignment to make this approach applicable to any dataset.

## Footnotes

1 faster_rcnn_inception_resnet_v2_atrous_oid.

2 POS tagger in spaCy v2: en_core_web_md from https://spacy.io/models/en.

3 We note that these embeddings will be different from those learned for all input words as part of the MT model training. Another option would have been to use an empty vector, but in initial experiments this strategy performed worse.

4 https://github.com/lium-lst/nmtpytorch.
